# Perspectives of Registered Dietitian Nutritionists on Adoption of Telehealth for Nutrition Care during the COVID-19 Pandemic

**DOI:** 10.3390/healthcare9020235

**Published:** 2021-02-23

**Authors:** Cory Brunton, Mary Beth Arensberg, Susan Drawert, Christina Badaracco, Wendy Everett, Sharon M. McCauley

**Affiliations:** 1 Abbott Nutrition Division of Abbott, Columbus, OH 43219, USA; mary.arensberg@abbott.com (M.B.A.); susan.drawert@abbott.com (S.D.); 2 Avalere Health, Washington, DC 20005, USA; cbadaracco@avalere.com (C.B.); weverett@avalere.com (W.E.); 3 Academy of Nutrition and Dietetics, Chicago, IL 60606, USA; smccauley@eatright.org

**Keywords:** SARS-CoV-2, COVID-19, telehealth, telemedicine, telenutrition, malnutrition, registered dietitian nutritionists, Malnutrition Quality Improvement Initiative (MQii), challenges and solutions using telehealth for nutrition care

## Abstract

Widespread transmission of the severe acute respiratory syndrome coronavirus 2 (SARS-CoV-2) infection has resulted in a global coronavirus disease 2019 (COVID-19) pandemic that is straining medical resources worldwide. In the United States (US), hospitals and clinics are challenged to accommodate surging patient populations and care needs while preventing further infection spread. Under such conditions, meeting with patients via telehealth technology is a practical way to help maintain meaningful contact while mitigating SARS-CoV-2 transmission. The application of telehealth to nutrition care can, in turn, contribute to better outcomes and lower burdens on healthcare resources. To identify trends in telehealth nutrition care before and during the pandemic, we emailed a 20-question, qualitative, structured survey to approximately 200 registered dietitian nutritionists (RDNs) from hospitals and clinics that have participated in the Malnutrition Quality Improvement Initiative (MQii). RDN respondents reported increased use of telehealth-based care for nutritionally at-risk patients during the pandemic. They suggested that use of such telehealth nutrition programs supported positive patient outcomes, and some of their sites planned to continue the telehealth-based nutrition visits in post-pandemic care. Nutrition care by telehealth technology has the potential to improve care provided by practicing RDNs, such as by reducing no-show rates and increasing retention as well as improving health outcomes for patients. Therefore, we call on healthcare professionals and legislative leaders to implement policy and funding changes that will support improved access to nutrition care via telehealth.

## 1. Introduction

The ongoing severe acute respiratory syndrome coronavirus 2 (SARS-CoV-2) outbreak has triggered the coronavirus disease 2019 (COVID-19) pandemic and caused a significant strain on medical institutions, particularly in the acute care setting. This disruption of healthcare services has multiple contributing factors: limited availability of adequate personal protective equipment (PPE), the need for defined populations to stay at home, and the high risk of infection spread among patients and healthcare professionals at care sites [1]. In order to follow the Centers for Disease Control and Prevention (CDC) guidelines on social distancing and limited group gatherings [2,3], adoption of telehealth—including telemedicine—has increased rapidly [4].

While the terms “telemedicine” and “telehealth” are sometimes used interchangeably [5], our paper distinguishes between them in several ways. Telemedicine refers to the use of technologies and telecommunication systems to provide clinical healthcare services to patients who are geographically separated from providers. For example, this could involve use of an Electronic Intensive Care Unit (eICU) or Tele-ICU, through which remote intensivists diagnose and treat critical patients in rural areas via technology such as videoconferencing. Telemedicine has facilitated specialty care for patients who live in distant, often rural, locations [6]. Telehealth is a broader term that can refer to clinical as well as non-clinical services. It also applies to the use of electronic information and telecommunications technologies to cover multiple health consultation activities—health monitoring and services, public health and health administration activities, and health education for patients and professionals. In this paper, we will refer to telehealth. Telehealth tools include video conferencing, e-mail, mobile or app-enabled technology, and technologies that transmit clinical information (data, image, audio, and video) [7]. 

About 11% of US consumers used telehealth in 2019 compared to an estimated 46% of consumers in 2020 that were often used to replace in-person visits [8]. Opinions about telehealth changed, as well. McKinsey reports 76% of consumers are interested in using telehealth going forward and 57% of providers view telehealth more favorably now than before the pandemic [8]. Researchers estimate telemedicine visits represented $29.3 billion of medical services in 2020 [4]; by 2023, up to $250 billion of US healthcare spending could be for virtual visits [4]. Telehealth may help increase efficiency in healthcare, too; prior to COVID-19, telehealth used with chronic disease populations was found to reduce total cost of care by 2–3% [8]. However, expanding telehealth also imposes costs on providers, such as through investment in infrastructure, workflow modifications and staff training, and malpractice and liability insurance [9]. Traditionally, telehealth visits have been reimbursed at a lower rate [10]. Thus, it is not surprising that adequate reimbursement has been identified as an important factor for enabling broad telehealth adoption [10]. 

With the onset of the COVID-19 pandemic, the US Department of Health and Human Services (HHS) declared a public health emergency that temporarily relaxed the existing restrictions on telehealth care [11]. For example, Health Insurance Portability and Accountability Act (HIPAA)-covered providers may, in good faith, provide telehealth services to patients using remote communication technologies (including commonly used applications such as FaceTime, Facebook Messenger, Google Hangouts, Zoom, or Skype) for telehealth services, even if the application does not fully comply with HIPAA rules [11]. Accordingly, the Centers for Medicare and Medicaid Services (CMS) also temporarily expanded access to make it easier for people enrolled in Medicare, Medicaid, and the Children’s Health Insurance Program (CHIP) to access medical services via telehealth during the pandemic [12]. CMS expansion currently allows use of telehealth services for: (i) patients located in their homes but outside of designated rural areas, (ii) remote care, even across state lines, (iii) care for new or established patients, and (iv) billing for telehealth services (video- or audio-only) as if they were provided in-person [11]. With these expansions enabling care during the COVID-19 pandemic, more than 9 million Medicare beneficiaries received a telehealth service between mid-March and mid-June 2020 [13]. 

Notably, telehealth services have also been applied to providing nutrition care for patients [7,14,15,16]. The Academy of Nutrition and Dietetics has defined this as telenutrition, involving the interactive use by a registered dietitian nutritionist (RDN) of electronic information and telecommunications technologies to implement the Nutrition Care Process with patients or clients at a remote location, within the provisions of the RDN’s state license as applicable [17]. Based on work with hospitals, clinics, and systems participating in the Malnutrition Quality Improvement Initiative (MQii) [18], we sought to better understand how RDNs were able to care for their patients during the COVID-19 pandemic, with particular focus on the extent to which telehealth services were being used and potential solutions to the barriers that both RDNs and patients faced. 

## 2. Background

Launched in 2013 by the Academy of Nutrition and Dietetics (Academy), Avalere Health, and other stakeholders, the MQii has established and supported a Learning Collaborative among more than 290 US hospitals and clinics to implement and disseminate best practices for malnutrition care [12,19,20,21]. With the onset of the COVID-19 pandemic, the MQii leadership team was interested in understanding how well RDNs at MQii member sites were able to meet the nutritional needs of their hospitalized patients, including those with and without COVID-19. To identify barriers faced by nutrition staff, we emailed an initial set of 14 questions to RDNs in the MQii Learning Collaborative [22]. In response to this survey, some RDNs described creative solutions they had developed, including the use of telehealth applications with fellow nutrition colleagues, physicians, nursing colleagues, and patients and their families.

With telehealth identified as a way to comply with physical restrictions and provide safe nutrition care for COVID-19 outpatients, we developed and disseminated a second qualitative, structured survey via email between 24 August and 11 September 2020. In this survey, we sought to obtain more information about how RDNs and teams were using telehealth to continue providing quality nutrition care to all patients (ICU, inpatient, and outpatient) during the pandemic. The 20-question survey (see Supplementary Material) was emailed to approximately 200 RDNs whose institutions participate in the MQii. This survey focused on RDN use of telehealth both before and during the pandemic. Responses were anonymous unless the RDN chose to add his/her name to be contacted for more information about the reported telehealth experience. We held follow-up structured interviews by telephone with six respondents who provided their contact information. 

## 3. Feedback from the Field

We received responses from 22 RDNs (19 from RDNs in clinical practice and three from food and nutrition service managers). Twenty of the hospitals had treated COVID-19 patients (with a case range of 90 to 1000) and two had not treated COVID-19 patients. Of the 22 respondents, 14 worked in academic medical centers and 8 worked in community hospitals. The size of the hospitals ranged from 34 to 1400 beds, with the majority having between 400 and 900 beds. All were general acute care hospitals with one exception of a rehabilitation facility. Twelve hospitals were in urban settings, one in a rural environment, and seven in a combination of urban and rural or suburban. All but two hospitals were part of integrated health systems. 

Six respondents reported that their hospitals had provided nutrition care via telehealth prior to the pandemic, 15 had not provided nutrition care via telehealth, and one did not know. During the pandemic, 20 respondents reported their hospitals are now providing nutrition telehealth services and 2 are not. The 20 RDNs and/or teams currently utilizing telehealth reported using it to provide the following services:

1. Nutrition education
2. Nutrition counseling
3. Nutrition care plan development
4. Nutrition assessment
5. Recommendations for nutrition supplementation
6. Nutrition discharge planning
7. Nutrition screening

Respondents reported using various telehealth modalities—live video conferencing, phone calls, web-based portals, and remote patient monitoring—during the COVID-19 pandemic. The largest increase in telehealth use by individual RDNs during COVID-19 occurred in telephone calls (15 RDNs).

Responses offered insights into positive perceptions, areas for improvement, and potential solutions for future support of nutrition care delivery via telehealth (Table 1). RDN respondents commonly reported that telehealth had positive effects on overall nutrition care in their hospitals/systems and rehab facility. Some reported plans to permanently adopt telehealth for nutrition care after the pandemic is over.

## 4. Discussion

While it is too early to know the long-term impacts of the COVID-19 pandemic on the US healthcare delivery system, our survey results give us early information about how telehealth can enhance nutrition care. Although the number of our responses was small, we were able to draw on the reported experiences of the MQii Learning Collaborative hospitals and other clinicians to identify opportunities that may help promote quality nutrition care via telehealth and better prepare for future challenges to their practice. The COVID-19 pandemic of 2020 has expedited the adoption of telehealth by many health systems. Information from our survey indicates that telehealth can be an acceptable alternative to in-person clinic and even inpatient visits. In fact, telehealth can enable healthcare providers to maintain usual caseloads and provides patients with uninterrupted healthcare access. Furthermore, telehealth offers opportunities for evaluation and education that might not exist in the face-to-face visit system. For example, telehealth visits have given RDNs the opportunity for longer assessment time with patients and the ability to “look in” their home environments to potentially observe their refrigerators and pantries, allowing further examination of their diet and nutrition habits. In other studies, telenutrition interventions have improved weight loss outcomes in cardiovascular disease patients in a pilot randomized controlled trial [23] and improved weight status in obese patients [24]. Telehealth has specifically been an effective resource for improving diabetes self-management in ethnically diverse and rural populations [25]. A systematic review and meta-analysis reported telenutrition for patients with chronic disease improved diet quality and dietary adherence when compared to face-to-face dietary counseling [26]. Being challenged by a crisis such as the COVID-19 pandemic presents an opportunity to create innovative nutrition solutions; telehealth is one of those.

Even before the current COVID-19 pandemic, Kelly et al. called for expanded use of telehealth for nutrition care and diet-related consultations for people living with a wide range of conditions, such as type 2 diabetes, cardiovascular disease, obesity, cancer, chronic kidney disease, and mental health conditions [16]. Their report presented evidence that dietitians could deliver high-quality, effective services by way of telehealth—notably, producing outcomes that are comparable to those resulting from in-person consultations [16]. In our survey, respondents identified one limitation of telehealth technology was not being able to conduct an NFPE, which is a key tool for generating evidence to diagnose malnutrition. From this observation, it seems that, when possible, in-person nutrition screening and assessment can best support malnutrition diagnosis and interventions, while telehealth may be better suited for ongoing patient monitoring and education in outpatient settings.

Mehta et al. specifically evaluated ways to apply telehealth-based nutrition care during the COVID-19 pandemic, including using telehealth for one-on-one RDN visits with existing outpatients [15]. They found that RDNs could obtain a thorough history and visualize a patient’s home environment [15], a finding that aligned with our survey findings. Mehta et al. also reported that group telehealth visits could efficiently deliver nutrition care. Group visits can include multiple family members of the same patient, a multidisciplinary group of caregivers with a patient, or several patients with one healthcare provider [15]. Group visits via telehealth allow for collaboration (such as the ability to virtually link patients, family members, primary caregivers, and consulting specialists) that is not otherwise possible. Previous research on family involvement in patient education has documented the effectiveness of family support for patient comprehension of nutrition interventions [27]. In our survey findings, family involvement was likewise suggested to enhance patient education, adherence to dietary advice, appointment-making, and resolution of telehealth technical issues.

Another potential benefit of telehealth nutrition care is that it increases access to medical nutrition therapy (MNT) by removing barriers related to limited finances, travel, time, and physical function. More than 10 years ago, Busey and Michael identified a role for “new and growing electronic technologies” to make healthcare services more accessible and less costly to a wider range of patients [28]. Today, the majority of RDNs continue to practice in acute care (40%) vs. ambulatory care (14%) or other care settings, which can limit patients’ access to MNT and make care transitions more difficult; telehealth nutrition can help solve both challenges [29,30]. In addition, as more practitioners become engaged in nutrition focused quality improvement programs (QIPs), incorporating telehealth into QIPs targeting transitions of care and outpatient settings may also present an opportunity to improve patient outcomes [31].

Several barriers to telehealth for patients must still be overcome. Our respondents identified lack of access to needed technology and connectivity issues as limitations. Similarly, results of other studies have identified technical/connectivity issues and limited telecommunication skills as common patient barriers to telehealth [32]. While healthcare providers report overall positive experiences with telehealth [33], concerns such as technical difficulties, lack of resources or organizational support, and increased provider workload remain [14]. Our survey identified the need to have additional staff members to help comply with variable payment guidelines and complete extra work to confirm insurance coverage. Farid has also recommended that preparation for successful telenutrition should include planning for billing [34]. Reimbursement for telehealth nutrition is a long-standing issue, identified a decade ago as one of the key practice barriers for adoption of telehealth nutritional care [28]. In our survey, one respondent suggested designating one person to provide support for coverage/billing for all telehealth nutrition visits to help address this problem.

Additional barriers for providers still need to be addressed. Ready support from an IT team can promote learning and trouble-shooting for new applications, incorporation of video (rather than audio-only) for more effective telehealth consultations, and increased provider training for electronic communication [33]. Our survey responses identified support from IT as a key area that exists but needs improvement. Healthcare organizations need to be forward-thinking and support development of the telehealth visit process to best position providers and patients for success. For individual telehealth visits, it is also important to address patient and provider concerns promptly. Survey responses suggested starting the actual telehealth visit a few minutes early to make sure the provider and patient are able to connect, scheduling a call ahead of the telehealth visit to help the patient navigate the portal, pre-screening to determine if telehealth would be appropriate for the patient, and utilizing a “script” to maximize the RDN’s time during the telehealth visit.

Perhaps the most critical need to continue expanding access to nutrition telehealth services is advocating to policymakers that MNT is essential for comprehensive and effective patient care. Discussions about healthcare reform need to consider all aspects of care that can be delivered effectively through telehealth means both during and beyond the COVID-19 pandemic. Medicare telehealth requirements that existed prior to the pandemic only covered nutrition services via live-video conferencing and under specific circumstances regarding the patient’s and practitioner’s physical locations. With the onset of the COVID-19 pandemic, CMS has lifted many of these restrictions [11], resulting in an drastic increase in patient telehealth visits [4]. Research from the University of Pennsylvania Perelman School of Medicine found that 67% of patients surveyed viewed their video and telephone appointments held during the peak of the COVID-19 pandemic as “positive and acceptable substitutes to in-person appointments” [35,36]. Our survey respondents reported positive patient experiences related to patients not needing to travel, find parking, sit in waiting rooms, take time off from work or childcare, and/or miss other appointments. It is well documented that the use of telehealth can also improve clinical outcomes, reduce costs [25,37,38], satisfy patients with their nutrition care, and leave individuals with positive perceptions/attitudes [39,40]. Further nutrition-specific research is needed to show such improved outcomes among different patient populations, including the comparison of patients between urban and rural areas. Access to technology—particularly for low-income patients, older patients, and those in rural areas—continues to be a concern for telehealth expansion and could prove a barrier for telenutrition, as well [9]. Changes to healthcare policy should focus on continuing to expand access to telehealth nutrition services by changing payment policies, allowing cross-state licensure of providers, and providing funding to underserved communities to increase their connectivity and access to telehealth services as well as other changes that expand MNT (Table 2).

To help address the barriers practitioners face in using telehealth and to share their learning, some organizations are already offering practical guidance on adopting telehealth practices while working within evolving regulatory frameworks. For example, the Academy of Nutrition and Dietetics launched its Telehealth Quick Guide for RDNs practicing via telehealth during the COVID-19 pandemic [41]. The MQii also shares COVID-19 resources with telehealth practice tips to guide multidisciplinary nutrition teams through its website and group calls [42]. The National Consortium of Telehealth Resources also provides assistance, education, and information to organizations and individuals who are actively providing or are interested in providing health care via telehealth and has developed a COVID-19 Telehealth Toolkit [43].

## 5. Recommendations

Our survey respondents reported positive experiences as well as challenges to using telehealth; they also shared recommendations that underscore a need for continued exploration of the patient, provider, economic, and policy implications of telehealth use. It is still unknown how the rapid and drastic switch to telehealth services during the pandemic will affect providers, payers, and patients financially as well as the percentage of visits that will ultimately return to in-person format. To advance health information technology policies and practices, further research should investigate how nutrition services via telehealth compares to other telehealth services as well as how to overcome barriers and identify the best path forward for providing effective and efficient nutrition care. This can ensure the appropriate patients have ready access to the care they need, regardless of where they reside. Continued research should also explore patient preferences and options for improving their reception to telehealth as well as determining the optimal applications and means for integrating with in-person visits for facilitating transitions of care. We call on healthcare professionals and legislative leaders alike to work together to extend policy and payment changes that will support nutrition care provided via telehealth as appropriate throughout and beyond the COVID-19 pandemic.

## Figures and Tables

**Table 1:** Summary of common positive perceptions, areas for improvement, and solutions suggested for providing nutrition care via telehealth.

Positive Perceptions	Areas for Improvement	Solutions Suggested
Allows Registered Dietitian Nutritionists (RDNs) to provide timely patient services while patients remain safe at home Provides RDNs with an opportunity to “look into” a patient’s refrigerator/pantry to better understand home environment and diet	Inability to conduct complete NFPE * via telehealth, especially if limited to audio-only consultation Inability to have direct patient contact, see patients face-to-face, or physically assess nutrition status	Pre-screen patients to determine if good candidate for telehealth visit Create guiding “scripts” for RDNs for optimal telehealth visits Send patient educational materials ahead of time to facilitate review during telehealth visit
Patients more likely to keep appointments Patients do not have to travel, find parking, or sit in waiting rooms	Telehealth nutrition payment guidelines remain unclear Care involves extra work to check each patient‘s health plan coverage Telehealth services may require additional staff	Designate one person to validate coverage/billing codes for all nutrition telehealth visits
Staff members able to continue seeing full caseload of outpatients during pandemic quarantine	Not all patients have access to technology needed for telehealth Lack of connectivity and/or patient-user skills limit care for some Lack of information technology (IT) departments’ support for high-quality RDN visits; technical issues with video/audio connections	Schedule call ahead of telehealth visit to walk patient through navigating portal (which IT can also support) With patient’s permission, engage family members to help with scheduling, technical issues, nutrition education, and patient adherence to recommendations

* NFPE: Nutrition-focused physical exam.

**Table 2:** National legislative and regulatory changes needed to support improved access to telehealth nutrition services.

Policy Area	Description of Policy Change Needed	Potential Impact
Public health emergency-driven telehealth flexibilities	Permanently waive originating and geographic site restrictions Allow for audio-only service provision when this is the only technology available Allow Medicare Advantage (MA) plans to make real-time changes that incorporate telehealth services into their benefit plans Allow cross-state licensure of various providers; this should include enforcing consistency in defining a “provider”	Expand MNT provision and access to all Medicare and MA beneficiaries who need it
Medical Nutrition Therapy (MNT) coverage	Reintroduce the MNT Act that broadens Medicare coverage of MNT services to include additional diseases and conditions, such as malnutrition and cancer; also expands providers who can refer patients for MNT to include nurses and other allied health professionals	

## Data Availability

Restrictions apply to the availability of these data. Data was obtained from Avalere Health and Abbott Nutrition and are available from the authors with the permission of Avalere Health and Abbott Nutrition.

## Supplementary Materials

The following are available online at https://www.mdpi.com/2227-9032/9/2/235/s1, Figure S1: Nutrition Care and Telehealth Related to the COVID-19 Pandemic and Beyond Survey
