# Supplementary material for: Perspectives of Registered Dietitian Nutritionists on Adoption of Telehealth for Nutrition Care during the COVID-19 Pandemic
Source: Healthcare (Basel). 2021 Feb 23;9(2):235. doi: 10.3390/healthcare9020235 (PMC7926532; doi:10.3390/healthcare9020235)
Supplement: Supplementary file 1 [file healthcare-09-00235-s001.pdf]

Figure S1. Nutrition Care and Telehealth Related to the COVID-19 Pandemic and Beyond Survey.

Thank you for taking the time to complete this survey – the information provided will be extremely valuable in advancing nutrition care.

Objectives

- Determine how providers were using telehealth prior to the COVID-19 pandemic (prior to February 1, 2020) and how their telehealth practices may have evolved and changed over time this year
- Identify positive/negative impacts and future implications for using telehealth in nutrition care

For this questionnaire, Telehealth refers to team consultation and conferencing, delivery of clinical services, education, patient monitoring and follow-up. It does not refer to patient scheduling or account management processes.

1. What is your current clinical designation?
    a. ___________ Registered Dietitian Nutritionist
    b. ___________ Registered Nurse
    c. ___________Physician
    d. ____________Administrator
    e. ____________Other (please specify)

2. Has your institution treated any COVID-19 patients since February 1, 2020?

________________ YES

_________________NO

If yes, approximately how many cases of COVID-19 has/have your nutrition department(s) treated between February 1, 2020 and today?

____

If no, please continue to work through the questionnaire. All responses are valuable.

TELEHEALTH AND YOUR INSTITUTION (Health system, organization, hospital or facility, including, acute care, rehabilitation, academic medical center, etc.)

3. What is your experience with telehealth prior to and post COVID-19 pandemic (February 1, 2020)? Please put an X in the appropriate box.

	Yes	No	Don't Know
Prior to the pandemic, did your **institution** provide access to telehealth?			
Post pandemic, does your **institution** now provide access to telehealth?			
Prior to the pandemic, had **nutrition care** been delivered to patients via telehealth?			
Post pandemic, is there now access to **nutrition care** for patients via telehealth?			
Prior to the pandemic, had you **personally** used a form of telehealth to deliver patient care?			
Post pandemic, have you **personally** used a form of telehealth to deliver patient care?			

4. If you responded with any "yes" in question 2, what forms of telehealth have your institution and/or you used for provision of clinical services? Place an X in all that apply.

**Telehealth Service**	**Institution Has Used**		**You Have Used**	
	**Prior to COVID-19**	**Post COVID-19**	**Prior to COVID-**	**Post COVID-19**
Live video conferencing				
Phone calls				
Web-based portals				
Remote patient monitoring				
Other (specify)				
Not applicable				

5. Have your telehealth platforms (technologies) changed over time since the start of the pandemic (February 1, 2020)?

____________ No

____________ Yes

Prior Technology: ____________________ (e.g. FaceTime)

Current Technology: ____________________(e.g. Zoom)

6. Is telehealth allowing you to perform your job duties effectively? Why or why not?

7. What were patients/families' reactions to telehealth prior to the COVID-19 pandemic and after? Please put an X in the appropriate box to indicate your answer

	They like it and use it	They are indifferent towards it	They do not like it or want to use it	Don't know
1. Prior to COVID-19				
2. After COVID-19				

8. Do you anticipate that your institution might permanently adopt the changes it has implemented in telehealth after the pandemic has resolved?

_________________Yes
_________________No
_________________No changes implemented
_________________Don't know

9. Why or why not might your institution permanently adopt the changes it has implemented in telehealth?

TELEHEALTH AND NUTRITION CARE

10. What types of nutrition care have your team and/or you been able to provide via telehealth? Check all that apply.

**Telehealth Service**	**Team Provides via Telehealth**	**You Provide via Telehealth**
Nutrition screening		
Nutrition assessment		
Nutrition care plan development		
Nutrition counseling		
Nutrition education		
Nutrition discharge planning		
Nutrition supplementation recommendation		
Other (specify)		
Not applicable		

11. Has telehealth impacted the overall nutrition care provided to COVID-19 patients <u>and</u> non-COVID-19 patients in your institution?

    _______Yes

    _______No

12. If yes, how?

13. What are some positive impacts nutrition care via telehealth is having for you, your colleagues and/or patients/families?

14. What are the most important challenges/negative impacts that you have frequently encountered in providing quality nutrition care to your patients via telehealth?

15. What workarounds or alternative care approaches have you and/or your team implemented in response to these challenges?

16. What improvements could be made to increase the effectiveness of nutrition care given via telehealth?
17. **GENERAL INFORMATION**

18. Please describe your healthcare institution.

**Characteristic**	**Your Response**
Academic teaching institution (yes/no)	
Size/Number of patient beds	
Specialty institution (if yes, specify)	
Urban or rural	
Part of health system (yes/no)	

19. We would appreciate any other information or observations you may have about the response of institutional nutrition departments to the use of telehealth during the COVID-19 pandemic. If you would be willing to have a short follow-up conversation with one of our team members, please check yes below and include your name, email, and phone number. You may also add further comments below.

________________No Thanks

_______________Yes Name _______________________________

Institution _______________________________

Phone Number _______________________________

Email Address ______________________________

Thank you!
